# Primary mediastinal large B-cell lymphoma (PMLBCL): long-term results from a retrospective multicentre Italian experience in 138 patients treated with CHOP or MACOP-B/VACOP-B

**DOI:** 10.1038/sj.bjc.6601460

**Published:** 2004-01-20

**Authors:** G Todeschini, S Secchi, E Morra, U Vitolo, E Orlandi, F Pasini, E Gallo, A Ambrosetti, C Tecchio, C Tarella, A Gabbas, A Gallamini, L Gargantini, M Pizzuti, G Fioritoni, L Gottin, G Rossi, M Lazzarino, F Menestrina, M Paulli, M Palestro, M G Cabras, F Di Vito, G Pizzolo

**Affiliations:** 1 Department of Hematology, Verona University, Italy; 2 Department of Hematology, Ospedale Niguarda, Milano, Italy; 3 Department of Hematology, Ospedale S. Giovanni Battista, Torino, Italy; 4 Department of Hematology, Pavia University, Italy; 5 Department of Oncology, Verona University, Italy; 6 Department of Hematology, Torino University, Italy; 7 Department of Hematology, Ospedale S. Francesco, Nuoro, Italy; 8 Department of Hematology, Ospedale Santa Croce, Cuneo, Italy; 9 Department of Hematology, Ospedale S. Carlo, Potenza, Italy; 10 Department of Hematology, Ospedale Santo Spirito, Pescara, Italy; 11 Anesthesiology and Intensive Care Institute, Verona University, Italy; 12 Department of Hematology, Spedali Riuniti, Brescia, Italy; 13 Department of Pathology, Verona University, Italy; 14 Department of Pathology, Pavia University, Italy; 15 Department of Pathology, Torino University, Italy; 16 Department of Hematology, Ospedale Businco, Cagliari, Italy; 17 Department of Oncology, Ospedale Regionale, Aosta, Italy

**Keywords:** PMLBCL, CHOP, MACOP-B/VACOP-B

## Abstract

The optimal treatment of primary mediastinal large B-cell lymphoma (PMLBCL) is still undefined. In the absence of randomised studies, we retrospectively analysed: (a) the effectiveness of two chemotherapy regimens (CHOP *vs* MACOP-B/VACOP-B) in complete remission (CR) achievement and event-free survival (EFS) and (b) the role of mediastinal involved-field radiotherapy (IF-RT) as consolidation. From 1982 to 1999, 138 consecutive patients affected by PMLBCL were treated in 13 Italian institutions with CHOP (43) or MACOP-B/VACOP-B (95). The two groups of patients were similar as regard to age, gender, presence of bulky mediastinal mass, pleural effusion, stage and international prognostic indexes category of risk. Overall, 75.5% of patients in CR received IF-RT as consolidation. Complete remission was 51.1% in the CHOP group and 80% in MACOP-B/VACOP-B (*P*<0.001). Relapse occurred in 22.7% of CHOP- and in 9.2% of MACOP-B/VACOP-B-treated patients (n.s.). Event-free patients were 39.5% in CHOP and 75.7% in the MACOP-B/VACOP-B group (*P*<0.001). The addition of IF-RT as consolidation improved the outcome, irrespectively of the type of chemotherapy (*P*=0.04). At a multivariate analysis, achievement of CR (*P*<0.0001) and type of CT (MACOP-B/VACOP-B) retained the significance for OS (*P*=0.008) and EFS (*P*=0.03). In our experience, MACOP-B/VACOP-B appears to positively influence OS and EFS in patients affected by PMLBCL, as compared to CHOP. Consolidation IF-RT on mediastinum further improves the outcome of CR patients.

PMLBCL is a subtype of diffuse large B-cell lymphoma (DLCL), recently recognised as a clinical–pathological entity (Harris *et al*, 1994). It accounts for a percentage between 6 and 13% of DLCLs (Falini *et al*, 1995; Cazals-Hatem *et al*, 1996).

PMLBCL histology is characterised by a diffuse proliferation of large B cells with prominent, roundish nuclei, clear cytoplasm and high proliferative index. A thin band of sclerosis often compartmentalises neoplastic elements (Menestrina *et al*, 1986). Lymphoma cells are CD45, CD20, CD19 and CD22 positive, while CD5, CD10, CD21 and HLA-DR are usually absent (Yousem *et al*, 1985; Addis and Isaacson, 1986; Moller *et al*, 1987; Haioun *et al*, 1989; Lamarre *et al*, 1989; Al-Sharabati *et al*, 1991; van Besien *et al*, 2001). Expression of CD30 is often present but weak. Surface and cytoplasmic immunoglobulins are rarely expressed. The thymic origin of the neoplastic B cells has been proven (Scarpa *et al*, 1987; Lamarre *et al*, 1989; Al-Sharabati *et al*, 1991; Rodriguez *et al*, 1994).

The high proliferation rate of neoplastic cells (Todeschini *et al*, 1990; Falini *et al*, 1995) correlates with the rapid growth and with the frequent presence of necrotic areas in the lymphoma mass (Aisenberg, 1999). Sclerosis can explain the frequent persistence of variable degrees of residual mass even after successful treatment (Rohatiner *et al*, 1994).

PMLBCL affects young people with a female prevalence (Todeschini *et al*, 1990; Lazzarino *et al*, 1993; Falini *et al*, 1995). Its rapid growth explains the frequent bulky mediastinal mass infiltrating thoracic structures (pericardium, pleura) and the chest wall. Vessels are frequently involved, leading to Superior Vena Cava Syndrome (SVCS) and to thrombosis (Perrone *et al*, 1986; Lamarre *et al*, 1989; Todeschini *et al*, 1990). Other symptoms at the onset are chest pain, cough and dyspnea (Lamarre *et al*, 1989; Todeschini *et al*, 1990; Al-Sharabati *et al*, 1991; Falini *et al*, 1995). Most cases are stage I–II. When the lymphoma spreads beyond the diaphragm, it usually involves the kidney and suprarenal glands (Perrone *et al*, 1986; Haioun *et al*, 1989; Todeschini *et al*, 1990; Kirn *et al*, 1993; Lazzarino *et al*, 1993). Bone marrow involvement is extremely rare (Cazals-Hatem *et al*, 1996; Abou-Ella *et al*, 1999). Diagnosis is mostly obtained by thoracotomy or mediastinoscopy, superficial lymph nodes being rarely involved.

Most published reports on PMLBCL mainly focus on the clinical–pathological features, and few papers only analyse the role of different chemotherapy regimens on the clinical outcome. As a result, the optimal therapy has not been defined yet, as pointed out by a recent review (van Besien *et al*, 2001).

According to literature, CHOP and third-generation regimens, followed or not by involved-field radiotherapy (IF-RT), are the most employed combination therapies (Levitt *et al*, 1982; Jacobson *et al*, 1988; Todeschini *et al*, 1990; Lazzarino *et al*, 1993; Falini *et al*, 1995; Cazals-Hatem *et al*, 1996; Martelli *et al*, 1998; van Besien *et al*, 2001).

A possible superiority of third-generation regimens in DLCLs has not been validated by a large prospective randomised study (Fisher *et al*, 1993). However, this study was not specifically focused on PMLBCLs. By contrast, data from other series suggest that, in this setting, third-generation therapies (usually MACOP-B and VACOP-B) may be more effective than CHOP (Levitt *et al*, 1982; Todeschini *et al*, 1990; Bertini *et al*, 1991; Falini *et al*, 1995; Cazals-Hatem *et al*, 1996; Bieri *et al*, 1999; van Besien *et al*, 2001; Zinzani *et al*, 2001, 2002).

In absence of randomised trials, we retrospectively analysed a large series of PMLBCL consecutive patients treated with CHOP or MACOP-B/VACOP-B observed in 13 different Italian institutions.

The first aim of this analysis was to compare the long-term results of CHOP *vs* MACOP-B/VACOP-B, the two most commonly employed regimens in PMLBCL patients in USA and Europe, respectively (van Besien *et al*, 2001). Patients treated with other regimens and those receiving stem cell transplant (SCT) as consolidation were not analysed. The two therapeutic approaches have been compared, taking into account the recognised international prognostic indexes (IPI) (The International Non-Hodgkin's Lymphoma Prognostic Factors Project, 1993).

The second objective of our analysis was to evaluate the role of consolidation IF-RT on the long-term outcome after CR achievement.

This paper reports our multicentre retrospective experience.

## PATIENTS AND METHODS

### Patients

Our review includes 138 consecutive patients observed in 13 Italian institutions between 1982 and 1999. Patients received routine staging procedures (complete physical examination, laboratory tests including serum LDH and serum beta-2 microglobulin, chest X-ray, CT scan of the chest, abdomen and pelvis, bone marrow aspirate, and biopsy). Stage was defined according to the Ann Arbor staging system. Bulky disease was defined as a mediastinal mass larger than 1/3 of thoracic diameter or a mediastinal mass larger than 10 cm (major diameter).

### Diagnostic criteria

All biopsy specimens were reviewed and confirmed according to the WHO criteria for PMLBCL diagnosis. Immunopheno-typical analysis was always performed. Other NHLs involving the mediastinum and not fulfilling these criteria were not considered.

### Selection criteria

Only patients treated with CHOP or MACOP-B/VACOP-B were included in this report. Patients who underwent other regimens or patients who received SCT after completing CHOP or MACOP-B/VACOP-B were excluded. Each institution chose treatment (CHOP or MACOP-B/VACOP-B) according to the local policy at the time. Chemotherapy was based on six cycles of CHOP or on a full (12 weeks) cycle of MACOP-B/VACOP-B. In each group of therapy, patients were divided into low/low-intermediate- and high-intermediate/high-risk groups according to IPI.

Involved-field radiotherapy was administered in patients in complete remission (CR)/near-complete remission (NCR). The decision to employ IF-RT or not was made according to the local policy of each centre. The modality of IF-RT administration was the following: (a) when the mass involved only the mediastinum, the whole original disease was irradiated; (b) when the mass spread beyond the mediastinum displacing the lung, IF-RT was administered to involve the mediastinum volume plus 1.5 cm outside its borders. The median dosage of IF-RT was 34 Gy (range 30–45). Only patients who underwent IF-RT after achieving CR or NCR were considered for IF-RT significance on disease outcome.

### Response criteria

CR was defined as the disappearance of lymphoma lesions and resolution of symptoms for at least 3 months after the end of CT. Near-complete remission (i.e. unconfirmed CR) was defined as the reduction of mass (lymphoma lesions) >90%, without new growth at physical and imaging examination, without signs and symptoms of disease for at least 3 months. Partial remission (PR) was defined as reduction of at least 50% of the original measurable mass. No remission (NR) was defined as (a) minimal modification (a response less than 50%) or (b) no modification or (c) progression of the mass under CT or (d) new lymphoma growth during CT or within 3 months after the end of CT.

In our review, gallium scan could not be considered in the assessment of CR, since only a minority of patients underwent this procedure. The possible persistence of some degree of fibrosis after CT even in responders makes it difficult to define the real incidence of CR. However, the long-term observation of our study allowed us to clarify this point, since NR or low responsive cases had invariably a rapid fatal outcome. Therefore, the long-term EFS had been judged *a posteriori* as an indicator of CR achievement. OS was calculated from the beginning of chemotherapy to the death or the last follow-up. EFS was defined as survival from the beginning of chemotherapy, in the absence of unfavourable events such as (1) toxic death, (2) NR, PR or progression, (3) relapse and (4) death due to any cause.

### Statistical analysis

Statistical analysis was performed using the *χ*^2^ test with Yates correction and with Fisher's exact test for categorical variables. Survival curves were calculated from the beginning of the treatment according to the actuarial method proposed by Kaplan and Meier. Differences between curves were evaluated by log-rank test. Cox regression analysis was performed to determine the independent contribution of the following variables: achievement of CR, type of treatment (CHOP *vs* MACOP-B/VACOP-B) and IPI score (low/low-intermediate *vs* high-intermediate/high). Each variable was compared to the previous one.

## RESULTS

Among the 138 patients, there were 75 females and 63 males (F/M 1.19). The median age was 39 (range 14–70). Only seven out of 138 patients were over 60. Most patients were in stage I–II (95 out of 138: 68.9%); stage III–IV patients were 43 out of 138 (31.1%).

Bulky mediastinal mass was present in 111 out of 138 (80.4%) and bone marrow involvement in two out of 138 (1.4%) cases. In all, 15 patients in stage IV had organ involvement beyond the diaphragm; in 13 (86.6%) of them, renal and suprarenal glands involvement was present. A total of 43 patients have been treated with CHOP and 95 with MACOP-B/VACOP-B. According to IPI, the different risk groups were: low, 82 out of 138 (59.4%); low-intermediate, 25 out of 138 (18.1%); high-intermediate, 23 out of 138 (16.6%); high, eight out of 138 (5.7%). The two risk categories were balanced in CHOP- and MACOP-B/VACOP-B-treated groups (Table 1

**Table 1:** Clinical characters of patients treated with CHOP or MACOP-B/VACOP-B

	**CHOP**	**MACOP-B/VACOP-B**	***P***
Patients	43	95	
Median age	35 (17–70)	36.5 (14–65)	
Gender (F/M)	27/16 (1.68)	48/47 (1.02)	
B symptoms	9/43 (20.9%)	48/95 (50.5%)	0.002
Bulky mediastinum	32/43 (74.4%)	79/95 (83.1%)	n.s.
Pleural effusion	19/43 (44.1%)	43/95 (45.2%)	n.s.
Pericardial effusion	9/43 (20.9%)	28/95(29.4%)	n.s.
SVCS	25/43 (58.1%)	43/95 (45.2%)	n.s.
Stage I–II	29/43 (67.4%)	66/95 (69.4%)	n.s.
Stage III–IV	14/43 (32.5%)	29/95 (30.5%)	n.s.
IPI			
Low/low-intermediate	25/39 (64.1%)	62/92 (67.3%)	n.s.
High-intermediate/high	14/39 (35.8%)	30/92 (32.6%)	n.s.
Study period			
1982–1990	26 (60.5%)	45 (47.3%)	n.s.
1991–1999	17 (39.5%)	50 (52.6%)	n.s.
Median follow-up (censored patients)	78 mo (8–199+)	91.5 mo (10–172+)	

).

The median follow-up of all entered patients was 66.5 months (1–199+). The median follow-up of patients alive in CR was 89.5 months (8–199+): 78 months (8–199+) in CHOP- and 91.5 months (10–172+) in MACOP-B/VACOP-B-treated groups.

The overall CR rate was obtained in 98 out of 138 (70%) and event-free patients were 89 out of 138 (64.4%). Complete remission/NCR were 22 out of 43 (51.1%) in the CHOP group and 76 out of 95 (80%) in the MACOP-B/VACOP-B group (*P*<0.0001). No remission/PD were 18 out of 43 (41.8%) and nine out of 95 (9.4%), respectively (*P*<0.001). Event-free patients were 17 out of 43 (39.5%) in CHOP- and 72 out of 95 (75.7%) in MACOP-B/VACOP-B-treated groups (Table 2

**Table 2:** Overall results comparing CHOP to MACOP-B/VACOP-B

	**CHOP**	**MACOP-B/VACOP-B**	***P***
Patients	43	95	
CR/NCR	22/43 (51.1%)	76/95 (80%)	*P*<0.001
PR	3/43 (6.9%)	10/95 (10.5%)	n.s.
NR/PD	18/43 (41.8%)	9/95 (9.4%)	*P*<0.001
REL	5/22 (22.7%)	7/76 (9.2%)	n.s.
EFS	17/43 (39.5%)	72/95 (75.7%)	*P*<0.001

; Figure 1

**Figure 1:**
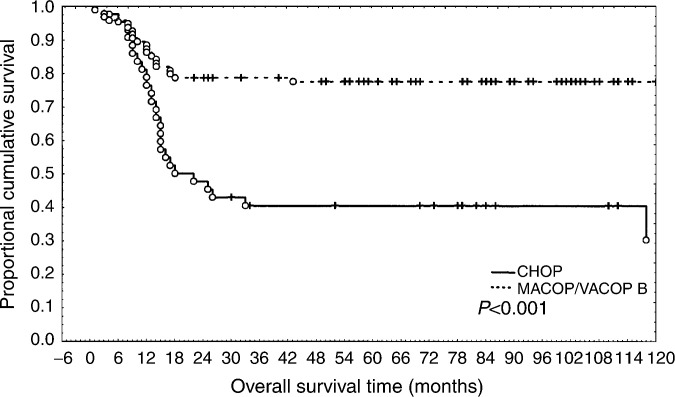
Event-free survival in CHOP- *vs* MACOP/VACOP-B-treated patients.

).

Considering IPI, in low/low-intermediate risk, 17 out of 33 (51.1%) CHOP-treated patients achieved CR, as opposed to 62 out of 74 (83.7%) MACOP-B/VACOP-B-treated ones (*P*=0.001). Event-free patients were 14 out of 33 (42.4%) and 60 out of 74 (81%), respectively (*P*=0.0001) (Table 3

**Table 3:** Results of CHOP *vs* MACOP-B/VACOP-B in 138 consecutive patients according to IPI

	**CHOP**	**MACOP-B/VACOP-B**	***P***
Low/low-intermediate risk			
Patients	33/43 (76.7%)	74/95 (77.8%)	n.s.
CR/NCR	17/33 (51.5%)	62/74 (83.7%)	*P*=0.001
PR	1/33	6/74 (8%)	n.s.
NR/PD	15/33 (45.4%)	6/62 (9.6%)	*P*=0.001
REL	3/17 (17.6%)	5/62 (8%)	n.s.
Event-free patients	14/33 (42.4%)	60/74 (81%)	*P*<0.001
			
High-intermediate/high risk			
Patients	10/43 (23.2%)	21/95 (22.1%)	n.s.
CR/NCR	5/10 (50%)	14/21 (66.6%)	n.s.
PR	2/10 (20%)	4/21 (19%)	n.s.
NR/PD	3/10 (30%)	3/21 (14.2 %)	n.s.
REL	2/5 (40%)	1/21 (4.7%)	n.s
Event-free patients	3/10 (30%)	12/21 (57.1%)	n.s.

). In the small group (31 out of 138, 22.4%) of high-intermediate/high-risk patients, five out of 10 (50%) CHOP-treated patients achieved CR *vs* 14 out of 21 (66.6%) MACOP-B/VACOP-B-treated ones (*P*=0.068).

Of the 37 patients in stage IV, only 17 (45.9%) became event-free survivors. Among the 15 patients with organ involvement beyond the diaphragm (four treated with CHOP, 11 with MACOP-B), only five (33.3%) were event-free (all treated with MACOP-B/VACOP-B). Relapses occurred after a median time of 9 months (range 3–77), mostly (eight out of 12: 66.6%) within 1 year from CR.

### Involved-field radiotherapy was considered for patients in CR/NCR

The majority of patients who achieved CR with CT received IF-RT on the mediastinum as consolidation (74 out of 98: 75.5%).

**Figure 2:**
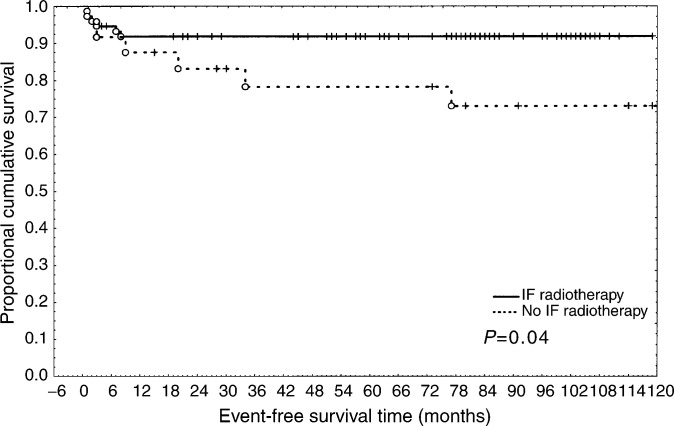
Event-free survival in CR patients treated or not with IF-RT.

Kaplan–Meier survival curve performed on the population who achieved CR showed a statistically significant difference in event-free survival time in patients receiving IF-RT (*P*=0.04) (Figure 2

).

Considering the achievement of CR, the type of treatment and the IPI levels as covariates, a multivariate Cox regression analysis for survival indicated that the achievement of CR and the type of treatment (MACOP-B/VACOP-B) were statistically significant. Differences between IPI levels did not achieve this condition (Table 4

**Table 4:** Multivariate analysis (Cox proportional hazards model) on 138 PMLBCL patients

**Covariates**	***P* value**	**Coefficient**	**95% CI**
Type of Chemotherapy (CHOP vs MACOP-B/VACOP-B)	0.027	0.498	0.269–0.923
CR achievement	0.000	11.507	5.378–24.624
IPI level	0.608	1.180	0.626–2.225

).

## DISCUSSION

Our analysis was focused on CHOP *vs* MACOP/VACOP-B, the most commonly regimens used in PMLBCL. The long-term results provided by this retrospective experience indicate that, in this setting, MACOP-B/VACOP-B achieve better results than CHOP, and confirm our previous experience (Todeschini *et al*, 1990). Additional retrospective data are in agreement with our observation (Bertini *et al*, 1991; Lazzarino *et al*, 1993; Falini *et al*, 1995; Cazals-Hatem *et al*, 1996; Lazzarino *et al*, 1997; Martelli *et al*, 1998; Bieri *et al*, 1999; Zinzani *et al*, 2001). Similar results were recently observed in a large retrospective study (Zinzani *et al*, 2002), which included patients treated with chemotherapy other than CHOP or MACOP/VACOP-B. Limiting the observations to CHOP and third-generation therapies, the authors found a projected 10-years progression-free survival (PFS) and OS of 35 and 44% with CHOP, and 67 and 71% with third-generation therapies, respectively.

Although a large randomised study (Fisher *et al*, 1993) showed no difference in outcome for intermediate-high-grade NHLs, in that study, the subgroup of PMLBCL was not separately considered.

In our series, advantages were statistically significant in the MACOP-B/VACOP-B-treated group at low/low-intermediate risk (*P*=0.001). In the small group of high-intermediate/high-risk patients, an advantage from MACOP-B/VACOP-B as compared to CHOP was observed in terms of CR rate and EFS, but it was not statistically significant (*P*=0.068).

Based on the results of our experience, we suggest that MACOP-B/VACOP-B can be more effective than CHOP in the treatment of PMLBCL. This assumption is supported by three observations.

First, although this is a retrospective review, the clinical characteristics of the two groups of patients were balanced (age, bulky mediastinum, pleural and pericardial effusion, presence of SVCS, stage I-II *vs* III-IV, type of chemotherapy during the study period) (Table 1).

Second, the IPI risk categories were equally distributed in the two therapeutic groups (Table 1).

Third, the two CT regimens were administered almost uniformly in the study time, thus avoiding the occurrence of possible biases due to different supportive measures.

The achievement of CR was the most significant prognostic factor both for OS and EFS (*P*<0.0001) (Table 4). A high percentage of patients who reached CR remained event-free, thus suggesting that a therapeutic approach able to increase the CR rate could influence the long-term OS and EFS. In fact, in both chemotherapy groups of our series, patients unresponsive or progressing under CT had invariably a fatal outcome (26 out of 26 died of lymphoma).

Relapse rarely affects PMLBCL patients, although it is the main cause of failure in other large-cell lymphomas. In our series, a very poor outcome was observed in the few relapsed patients (12.2%), since all of them died of lymphoma, independently of first-line therapy and subsequent salvage approaches (Table 2). Involved-field radiotherapy was ineffective in controlling the disease progression too. These findings are in agreement with the majority of reports (Moller *et al*, 1987; Jacobson *et al*, 1988; Cazals-Hatem *et al*, 1996; Zinzani *et al*, 2001), but contrast with the relatively good results described by other authors (Popat *et al*, 1998; Sehn *et al*, 1998) in small groups of refractory/relapsed patients treated with stem cell rescue.

In PMLBCL, the dismal outcome of relapsed/refractory patients seems even worser than in other DLCLs, and it appears as a further characteristic of this lymphoma entity. In fact, in other aggressive NHLs, a variable percentage of patients achieve a second CR and a small but not negligible proportion of them obtain a prolonged survival.

In our experience, patients with advanced stage of disease (i.e. subdiaphragmatic organ involvement) had a lower achievement of CR and a very poor outcome, suggesting that in these patients more intensive approaches, including stem cell transplantation, should be early considered.

Consolidation IF-RT is largely employed in PMLBCL patients, but its role is unclear. In our experience, the administration of IF-RT after CR achievement showed a positive impact on EFS, irrespective of the type of chemotherapy administered (*P*=0.04) (Figure 2). The favourable role of IF-RT is suggested by a recent large retrospective analysis (Zinzani *et al*, 2002).

The relative high percentage of NR in CHOP-treated patients and the extremely poor outcome of NR/relapsed patients suggest that the maximum effort should be done in the early phase of the disease, given the critical importance of the first-line therapy.

In fact, at the multivariate analysis, CR achievement and type of chemotherapy (MACOP-B, VACOP-B) were independent prognostic factors for EFS, while IPI did not result to be significant (Table 4).

According to these results and in the absence of randomised studies, MACOP-B/VACOP-B followed by IF-RT should be provisionally recommended for PMLBCL patients.

Randomised prospective studies are needed to define the optimal first-line chemotherapy and to assess the role of mediastinal IF-RT in PMLBCL treatment.

## References

[bib1] Abou-Ella AA, Weisenburger DD, Vose JM, Kollath JP, Lynch JC, Bast MA, Bierman PJ, Greiner TC, Chan WC, Armitage JO (1999) Primary mediastinal large B-cell lymphoma: a clinicopathologic study of 43 patients from the Nebraska Lymphoma Study Group. J Clin Oncol 17: 784–790 10071267 10.1200/JCO.1999.17.3.784

[bib2] Addis BJ, Isaacson PG (1986) Large cell lymphoma of the mediastinum: a B-cell tumor of probable thymic origin. Histopathology 10: 379–390 2423430 10.1111/j.1365-2559.1986.tb02491.x

[bib3] Aisenberg AC (1999) Primary large cell lymphoma of the mediastinum. Semin Oncol 26: 251–258 10375082

[bib4] Al-Sharabati M, Chittal S, Duga-Neulat I, Laurent G, Mazzerolles C, al-Saati T, Brousset P, Delsol G (1991) Primary anterior mediastinal B-cell lymphoma. A clinicopathological and immunohistochemical study of 16 cases. Cancer 67: 2579–2587 2015557 10.1002/1097-0142(19910515)67:10<2579::aid-cncr2820671030>3.0.co;2-h

[bib5] Bertini M, Orsucci L, Vitolo U, Levis A, Todeschini G, Meneghini V, Novero D, Tarella C, Gallo E, Luxi G (1991) Stage II large B-cell lymphoma with sclerosis treated with MACOP-B. Ann Oncol 2: 733–737 1724908 10.1093/oxfordjournals.annonc.a057853

[bib6] Bieri S, Roggero E, Zucca E, Bertoni F, Pianca S, Sanna P, Pedrinis E, Bernier J, Cavalli F (1999) Leuk Lymphoma 35: 537–544 10609791 10.1080/10428199909169618

[bib7] Cazals-Hatem D, Lepage E, Brice P, Ferrant A, d'Agay MF, Baumelou E, Briere J, Blanc M, Gaulard P, Biron P, Schlaifer D, Diebold J, Audouin J (1996) Primary mediastinal large B-cell lymphoma. A clinicopathologic study of 141 cases compared with 916 non-mediastinal large B-cell lymphomas, a GELA ("Groupe d'Etude des Lymphomes de l'Adulte") study. Am J Surg Pathol 20: 877–888 8669537 10.1097/00000478-199607000-00012

[bib8] Falini B, Venturi S, Martelli MSantucci A, Pileri S, Pescarmona E, Giovannini M, Mazza P, Martelli MF, Pasqualucci L, Ballatiori E, Guglielmi C, Amadori S, Poggi S, Sabattini E, Gherlinzoni F, Zinzani PL, Baroni CD, Mandelli F, Tura S (1995) Mediastinal large B-cell lymphoma. Clinical and immunohistological findings in 18 patients treated with different third-generation regimens. Br J Haematol 89: 780–789 7539625 10.1111/j.1365-2141.1995.tb08415.x

[bib9] Fisher RI, Gaynor ER, Dahlberg S, Oken MM, Grogan TM, Mize EM, Glick JH, Coltman CA, Miller TP (1993) Comparison of standard regimen (CHOP) with three intensive chemotherapy regimens for advanced non-Hodgkin's lymphoma. N Engl J Med 328: 1002–1006 7680764 10.1056/NEJM199304083281404

[bib10] Haioun C, Gaulard P, Roudot-Thoraval F, Divine M, Jouault H, Lebourgeois JP, Kuentz M, Farcet JP, Reyes F (1989) Mediastinal diffuse large-cell lymphoma with sclerosis. A condition with a poor prognosis. Am J Clin Oncol 12: 425–429 2801603 10.1097/00000421-198910000-00013

[bib11] Harris NL, Jaffe ES, Stein H, Banks PM, Chan JK, Cleary ML, Delsol G, de Wolf-Peeters C, Falini B, Gatter KC (1994) A revised European–American classification of lymphoid neoplasm: a proposal from the International Lymphoma Study Group. Blood 84: 1361–1391 8068936

[bib12] Jacobson JO, Aisenberg AC, Lamarre L, Willet CG, Linggood RM, Miketic LM, Harris NL (1988) Mediastinal large cell lymphoma. An uncommon subset of adult lymphoma curable with combined modality therapy. Cancer 62: 1893–1898 3167803 10.1002/1097-0142(19881101)62:9<1893::aid-cncr2820620904>3.0.co;2-x

[bib13] Kirn D, Mauch P, Shaffer K, Pinkus G, Shipp MA, Kaplan WD, Tung N, Wheeler C, Beard CJ, Cannellos GP (1993) Large-cell and immunoblastic lymphoma of the mediastinum. Prognostic and pathologic features in 57 patients. J Clin Oncol 11: 1336–1343 8315431 10.1200/JCO.1993.11.7.1336

[bib14] Lamarre L, Jacobson JO, Aisenberg AC, Harris NL (1989) Primary lymphoma of the mediastinum. A histologic and immunophenotypic study of 29 cases. Am J Surg Pathol 13: 730–739 2788371 10.1097/00000478-198909000-00002

[bib15] Lazzarino M, Orlandi E, Paulli M, Boveri E, Morra E, Brusamolino E, Kindl S, Rosso R, Astori C, Buonanno MC, Magrini U, Bernasconi C (1993) Primary mediastinal B-cell lymphoma with sclerosis. An aggressive tumor with distinctive clinical and pathologic features. J Clin Oncol 11: 2306–2313 8246020 10.1200/JCO.1993.11.12.2306

[bib16] Lazzarino M, Orlandi E, Paulli M, Strater J, Klersy C, Gianelli U, Gargantini L, Rousset MT, Gambacorta M, Marra E, Lavabre-Bertrand T, Magrini U, Manegold C, Bernasconi C, Moller P (1997) Treatment outcome and prognostic factors for primary mediastinal (Thymic) B-cell lymphoma: a multicenter study of 106 patients. J Clin Oncol 15: 1646–1653 9193365 10.1200/JCO.1997.15.4.1646

[bib17] Levitt LJ, Aisenberg AC, Harris NL, Linggood RM, Poppema S (1982) Primary non-Hodgkin's lymphoma of the mediastinum. Cancer 50: 2486–2492 7139540 10.1002/1097-0142(19821201)50:11<2486::aid-cncr2820501138>3.0.co;2-g

[bib18] Martelli MP, Martelli M, Pescarmona E, De Sanctis V, Donato V, Palombi F, Todisco E, Rensina EA, Pau FM, Mandelli F (1998) MACOP-B and involved field radiation therapy is an effective therapy for primary mediastinal large B-cell lymphoma with sclerosis. Ann Oncol 9: 1027–1029 9818079 10.1023/A:1008412009667

[bib19] Menestrina F, Chilosi M, Bonetti F, Lestani M, Scarpa A, Novelli P, Doglioni C, Todeschini G, Ambrosetti A, Fiore-Donati L (1986) Mediastinal large-cell lymphomas of B-type, with sclerosis: histopathological and immunohistochemical study of eight cases. Histopathology 10: 589–600 3525372 10.1111/j.1365-2559.1986.tb02512.x

[bib20] Moller P, Moldenhauer G, Momburg F, Lammler B, Eberlein-Gonska M, Kiesel S, Dorken B (1987) Mediastinal lymphoma of clear cell type is a tumor corresponding to terminal steps of B-cell differentiation. Blood 69: 1087–1095 3103712

[bib21] Perrone T, Frizzera G, Rosai J (1986) Mediastinal diffuse large cell lymphoma with sclerosis. A clinicopathologic study of 60 cases. Am J Surg Pathol 10: 176–191 3953939 10.1097/00000478-198603000-00005

[bib22] Popat U, Przepiork D, Champlin R, Pugh W, Amin K, Mehra R, Rodriguez J, Giralt S, Romaguera J, Rodriguez A, Preti A, Andersson B, Khouri I, Claxton D, de Lima M, Donato M, Anderlini P, Gajewski J, Cabanillas F, van Besien K (1998) High-dose chemotherapy for relapsed and refractory diffuse large B-cell lymphoma: mediastinal localization predicts for a favorable outcome. J Clin Oncol 16: 63–69 9440724 10.1200/JCO.1998.16.1.63

[bib23] Rodriguez J, Pugh WC, Romaguera JE, Luthra R, Hagemeister FB, McLaughlin P, Rodriguez MA, Swan F, Cabanillas F (1994) Primary mediastinal large cell lymphoma. Hematol Oncol 12: 175–184 8001905 10.1002/hon.2900120404

[bib24] Rohatiner AZ, Whelan JS, Ganjoo RK, Norton AJ, Wilson A, Lister TA (1994) Mediastinal large-cell lymphoma with sclerosis (MLCLS). Br J Cancer 69: 601–604 8123496 10.1038/bjc.1994.111 PMC1968881

[bib25] Scarpa A, Bonetti F, Menestrina F, Menegazzi M, Chilosi M, Lestani M, Bovolenta C, Zamboni G, Fiore-Donati L (1987) Mediastinal large-cell lymphoma with sclerosis. Genotypic analysis establishes its B-cell nature. Virchows Arch A. Virchows Arch A Pathol Anat Histopathol 412: 17–21 2825402 10.1007/BF00750725

[bib26] Sehn LH, Antin JH, Shulman LN, Mauch P, Elias A, Kadin ME, Wheeler C (1998) Primary diffuse large B-cell lymphoma of the mediastinum: outcome following high-dose chemotherapy and autologous hematopoietic cell transplantation. Blood 91: 717–723 9427731

[bib27] The International Non-Hodgkin Lymphoma Prognostic Factors Project (1993) A predictive model for aggressive non-Hodgkin's lymphoma. N Engl J Med 329: 987–994 8141877 10.1056/NEJM199309303291402

[bib28] Todeschini G, Ambrosetti A, Meneghini V, Pizzolo G, Menestrina F, Chilosi M, Benedetti F, Veneri D, Cetto GL, Perona G (1990) Mediastinal large B-cell lymphoma with sclerosis. A clinical study of 21 patients. J Clin Oncol 8: 804–808 1692089 10.1200/JCO.1990.8.5.804

[bib29] van Besien K, Kelta M, Bahaguna P (2001) Primary mediastinal B-cell lymphoma: a review of pathology and management. J Clin Oncol 19: 1855–1864 11251018 10.1200/JCO.2001.19.6.1855

[bib30] Yousem SA, Weiss LM, Warnke RA (1985) Primary mediastinal non Hodgkin's lymphomas. A morphologic and immunologic study of 19 cases. Am J Clin Pathol 83: 676–680 3923821 10.1093/ajcp/83.6.676

[bib31] Zinzani PL, Martelli M, Bendandi M, De Renzo A, Zaccaria A, Pavone E, Bocchia M, Falini B, Gobbi M, Gherlinzoni F, Stefoni V, Tani M, Tura S (2001) Primary mediastinal large B-cell lymphoma with sclerosis: a clinical study of 89 patients treated with MACOP-B chemotherapy and radiation therapy. Haematologica 86: 187–191 11224489

[bib32] Zinzani PL, Martelli M, Bertini M, Gianni AM, Devizzi L, Federico M, Pangalis G, Michels J, Zucca E, Cantonetti M, Cortelazzo S, Wotherspoon A, Ferreri AJM, Zaja F, Lauria F, De Renzo A, Liberati MA, Falini B, Balzarotti M, Calderoni A, Zaccaria A, Gentilini P, Fattori PP, Pavone E, Angelopoulou MK, Alinari L, Brugiatelli M, Di Rienzo N, Bonifazi F, Pileri SA, Cavalli F (2002) Induction chemotherapy strategies for primary mediastinal large B-cell lymphoma with sclerosis: a retrospective multinational study on 426 previously untreated patients. Haematologica 87: 1258–1264 12495899

